# Supplementary material for: Treatment Trends and Combined Methods in Removing Pharmaceuticals and Personal Care Products from Wastewater—A Review
Source: Membranes (Basel). 2023 Jan 27;13(2):158. doi: 10.3390/membranes13020158 (PMC9960457; doi:10.3390/membranes13020158)
Supplement: Supplementary file 1 [file membranes-13-00158-s001.zip › membranes-2145799-supplementary.pdf]

Supplementary Material

Treatment trends and combined methods in removing pharmaceuticals and personal care products from wastewater – a review

Paripurnanda Loganathan[1], Saravanamuthu Vigneswaran[1, 2, *], Jaya Kandasamy[1], Agnieszka Katarzyna[2], Zakhar Maletskyi[2], Harsha Ratnaweera[2]

[1] A Faculty of Engineering, University of Technology Sydney (UTS), P.O. Box 123, Broadway, NSW 2007, Australia
[2] Faculty of Sciences and Technology (RealTek), Norwegian University of Life Sciences, P.O. Box 5003, NO-1432 Ås, Norway
* Correspondence: Saravanamuth.vigneswaran@uts.edu.au

Table S1 Removal efficiency of PPCPs by biological treatment in wastewater treatment plants (WWTPs).

Compounds	Initial Conc (ng/l)	Treatment process	Removal Efficiency (%)	Reference
Hormone				
Estriol	60	Primary treatment + conventional activated sludge	66.8	[40]
Estrone	57		93.7	[40]
Antibiotics				
Sulfadiazine	20-22	Primary treatment + oxidation ditch + UV disinfection	40-100	[98]
Nonsteroidal anti-inflammatory drugs				
Ibuprofen	4500	Primary treatment + conventional activated sludge	99.7	[40]
	130-450	Primary treatment + oxidation ditch + UV disinfection	60-90	[98]
	20-70	Primary treatment + oxidation ditch + UV disinfection	10-60	[98]
Ketoprofen	70-220	Primary treatment + conventional activated sludge + tertiary treatment (ultrafiltration and ozonation)	0-80	[98]
Beta-blocker				
Atenolol	255	Primary treatment + primary sedimentation + bioreactor + clarifiers	47.1	[38]
Metoprolol	379	Primary treatment	52.9	[38]
Propanolol	151	Grit tanks + primary sedimentation + bioreactor + clarifiers	49.9	[38]
Antidepressant				
Fluxetine	51.1	Primary treatment + primary sedimentation + bioreactor + clarifiers	68.2	[38]

Modified from: Wang and Wang, [6].

Table S2 The removal of PPCPs by activated carbon.

Compounds	PAC Dose (mg/l)	Initial Conc (ng/l, except specified otherwise)	Source Water	q_m (mg/g)	Removal Efficiency (%)	Reference
Hormone						
Estriol	5	100	Surface water	n.a.	~60	[99]
Estrone	5	100	Surface water	n.a.	~72	[99]
Estradiol	5	100	Surface water	n.a.	~80	[99]
Antibiotics						
Sulfameth-oxazole	5	100	Surface water	n.a.	~35	[99]
	50	600	WWTPs effluents	n.a.	~60	[100]
Trimethoprim	5	100	Surface water	n.a.	~75	[99]
Liquid Regulator						
Bezafibrate	50	1.3 μg/l	WWTPs effluents	n.a.	~90	[100]
Gemfibrozi	5	100	Surface water	n.a.	~37	[99]
Nonsteroidal anti-inflammatory drugs						
Ibuprofen	5	100	Surface water	n.a.	~15	[99]
Diclofenac	5	100	Surface water	n.a.	~40	[99]
	50	5.8 μg/l	WWTPs effluents	n.a.	~80	[100]
Paracetamol	5	100	Surface water	n.a.	~70	[99]
Naproxen	5	100	Surface water	n.a.	~50	[99]
Antidepressant						
Diazepam	5	100	Surface water	n.a.	~65	[99]
Anticonvulsants						
Carbamazepine	5	100	Surface water	n.a.	~70	[99]

Modified from: Wang and Wang, [6].

Table S3 The removal of PPCPs by graphene and graphene oxide.

Compounds	Graphene Type	Initial Conc (mg/L)	Source Water	q_m (mg/g)	Removal Efficiency (%)	Reference
Antibiotics						
Sulfameth-oxazole	Graphene nanosheet (1 g/l)	100 - 200	Synthetic water	103	n.a.	[101]
	Graphene nanosheet (1 g/l)	100 - 200	Synthetic water	122	n.a.	[101]
Nonsteroidal anti-inflammatory drugs						
Ibuprofen	Graphene	10	Synthetic water	n.a.	95.5	[102]
Diclofenac	Graphene	10	Synthetic water	n.a.	97	[102]
Beta-blocker						
Atenolol	Graphene oxide (1 g/l)	60	Synthetic water	55.49	n.a.	[103]
Propanolol	Graphene oxide (1 g/l)	60	Synthetic water	42.48	n.a.	[103]
Carbamazepine	Graphene	10	Synthetic water	n.a.	97	[102]

Modified from: Wang and Wang, [6].

Table S4 Fenton oxidation of PPCPs.

Compounds	Initial conc.	Source water	Conditions	Removal efficiency (%)	References
Antibiotics					
Ofloxacin	0.0277 mmol/l	Waste water	25°C, pH2.8-3.2, 120 ming, Fe^{2+}- 5 mg/l, H_2O_2- 2.714 mM	100	104
Lipid regulator					
Bezafibrate	0.055 mM	Waste water	Photo-Fenton, room temp, pH2.5,10 min, Fe^{2+}- 0.2 mM, H_2O_2- 5.0 mM	100	105
Nonsteroidal anti-inflammatory drugs					
Ibuprofen	0.87 μM	Water	Fenton, 30°C, pH3, 2 h, Fe^{2+}- 1.2 mM, H_2O_2- 0.32 mM	>50	106
Diclofenac	0.17 mM	Ultrapure Water	25°C, pH3, 12 h, Pyrite- 0.5-4.0mM, H_2O_2- 1.0mM	>85	107
	33.4 mg/l	Distilled water/ Waste water	pH5-8, 15 min, ammonium Fe^{3+} citrate complex- 0.2mM, H_2O_2-68 mM	20-100	108
	50 mg/l	Demineral -ized water	20-40°C, pH7, Fe^{2+}- 0.05mM, H_2O_2- 200-400mg/l	100	109
Paracetamol	100 mg/l	Water	60°C, pH2.6, 5 h, magnetite- 6 g/l, H_2O_2- 28mM	100	110
Naproxen	20 mg/l	Water	28-33 °C, pH3, 10min, Fe^{2+}- 4.83 mg/l, H_2O_2-9.98 mM	100	111
Salicylic acid	4 mM	Double distilled water	20°C, pH3.0, 60 min, Fe^{2+}- 3.2 mM; H_2O_2- 12.8 mM	100	112
Beta Blocker					
Atenolol l	20 mg/l	Ultrapure Water	35°C, pH7, 150 min, Fe^{2+}- 5 mg/l, H_2O_2- 100 mg/l	100	113
Metoprolol	20 mg/l	Ultrapure Water	35°C, pH7, 150 min, Fe^{2+}- 2.8 mg/l, H_2O_2- 95 mg/l	100	113
Anticonvulsants					
Carbamazepine	42 μM	Double distilled water	21°C, pH5, 1 h, Fe_0- 10 mg H_2O_2- 10 ml	90	114
	100 mg/l	Waste water	Room temp, pH3, 180 min, Fe^{2+}-0.016 mM, H_2O_2-0.8 mM	>90	115
	60.35 μM	Ultrapure Water	23°C, pH7.0, 120 min, Fe_3O_4-1.0 g/l, H_2O_2-100mM	100	116

Modified from: Wang and Wang, [6].

Table S5 Photo-Fenton oxidation of PPCPs.

Compounds	Initial conc.	Source water	Conditions	Removal efficiency (%)	References
Antibiotics					
Tylosin	100 mg/l	Ultrapure water	Electro-Fenton, pH3.0, 15 min, Fe^{3+}- 0.1 mM, I- 300 mA	N.A	[117]
Enoxacin	50 mg/l	Ultrapure water	Electro-Fenton, 18°C, pH3.0, 60 min, Fe^{2+}- 0.2 mM, I- 300 mA	>90	[118]
Lipid regulator					
Clorfibric acid	179 mg/l	Pure Water	Electro-Fenton, 35°C, pH3.0, 12 min, Fe^{2+}- 1.0 mM, I- 100 mA	100	[119]
Nonsteroidal anti-inflammatory drugs					
Ibuprofen	0.87 mM	Water	Photo-Fenton, 30°C, pH3, 2 h, Fe^{2+}- 1.2 mM, H_2O_2- 0.32 mM	100	[106]
	100 mg/l	Synthetic water/ Simulated water/ Waste water	Photo-Fenton, 37°C pH7.6, 90 min, Fe^{2+} - 5 mg/l, H_2O_2 -50 mg/l	100	[120]
Diclofenac	50 mg/l	Demineral -ized water	20-40°C, pH7, Fe^{2+}- 0.05mM, H_2O_2- 200-400mg/l	100	[109]
Paracetamol	100 mg/l	Water	60°C, pH2.6, 5 h, magnetite- 6 g/l, H_2O_2- 28mM	100	[110]
Salicylic acid	4 mM	Double distilled water	Photo-Fenton, 20°C, pH3.0, 60 min, Fe^{3+}- 0.5 mM; H_2O_2- 10mM	82	[112]
Beta Blocker					
Metoprolol	0.246 mM	Ultrapure Water	Electro-Fenton, 35°C, pH3.0, 6 h, Fe^{2+}- 0.5 mM, Cu^{2+}- 0.1 mM, I- 120 mA	100	[121]
Anticonvulsants					
Carbamazepine	50 µM	Ultrapure Water	Photo-Fenton, 25°C, pH8.4, 30 min, Fe^{2+}- 0.1 mM, Persulphate- 0.2 mM	100	[122]

Modified from: Wang and Wang, [6].

Table S6 UV/hydrogen peroxide treatment of PPCPs in wastewaters.

Compounds	Initial conc.	Source water	Conditions	Removal efficiency (%)	References
Antibiotics					
Sulfamethoxazole	120 ng/l	Waste water	2768 mJ/cm^2; room temp, pH6.5, H_2O_2 - 1.72 g/l.15 min	100	[74]
	578 ng/l	Waste water	550 w/m^2, 17 ^oC, pH2.5, H_2O_2 - 50 mg/l, 30 min	100	[123]
Trimethoprim	~95 ng/l	Waste water	2768 mJ/cm^2; room temp, pH6.5, H_2O_2 - 1.72 g/l.15 min	100	[74]
	131 ng/l	Waste water	550 w/m^2, 17 ^oC, pH2.5, H_2O_2 - 50 mg/l, 30 min	100	[123]
Amoxicillin	25 mg/l	Distilled water	2.3 w/cm^2, 40 ^oC, pH7.0, H_2O_2 - 588 mg/l, 67 min, 10 rpm	~90	[124]
Erythromycin	110 ng/l	Waste water	2768 mJ/cm^2; room temp, pH6.5, H_2O_2 - 1.72 g/l,15 min	~98	[74]
Ofloxacin	41 ng/l	Waste water	550 w/m^2, 17°C, pH2.5, H_2O_2 - 50 mg/l, 30 min 100		[123]
Ciprofloxacin	129 ng/l	Waste water	550 w/m^2, 17°C, pH2.5, H_2O_2- 50 mg/l, 30 min	100	[123]
Tetracycline	~70 ng/l	Waste water	2768 mJ/cm^2; room temp, pH6.5, H_2O_2- 1.72 g/l.15 min	~99	[74]
Lipid regulator					
Bezafibrate	120 ng/l	Waste water	2768 mJ/cm^2; room temp, pH6.5, H_2O_2- 1.72 g/l, 15 min	100	[74]
	426 ng/l	Waste water	550 w/m^2, 17°C, pH2.5, H_2O_2 - 50 mg/l, 30 min	100	[123]
Gemfibrozil	25 ng/l	Waste water	550 w/m^2, 17°C, pH2.5, H_2O_2 - 50 mg/l, 30 min	>100	[123]
Nonsteroidal anti-inflammatory drugs					
Ibuprofen	112 ng/l	Waste water	550 w/m^2, 17°C, pH2.5, H_2O_2 - 50 mg/l, 30 min	100	[123]
Diclofenac	~90 ng/l	Waste water	2768 mJ/cm^2; room temp, pH6.5, H_2O_2 - 1.72 g/l,15 min	100	[74]
	518 ng/l	Waste water	550 w/m^2, 17°C, pH2.5, H_2O_2 - 50 mg/l, 30 min	100	[123]
Naproxen	178 ng/l	Waste water	550 w/m^2, 17°C, pH2.5, H_2O_2 - 50 mg/l, 30 min	100	[123]
Acetaminophen	~9 ng/l r	Waste water	2768 mJ/cm^2; room temp, pH6.5, H_2O_2 - 1.72 g/l.15 min	~90	[74]
Ketoprofen	100 ng/l	Waste water	2768 mJ/cm^2; room temp, pH6.5, H_2O_2 - 1.72 g/l.15 min	100	[74]
Carbamazepine	~95 ng/l	Waste water	2768 mJ/cm^2; room temp, pH6.5, H_2O_2 - 1.72 g/l.15 min	100	[74]
	263 ng/l	Waste water	550 w/m^2, 17 _C, pH2.5, H_2O_2 - 50 mg/l, 30 min	100	[123]

Primidone	~80 ng/l	Waste water	2768 mJ/cm^2; room temp, pH6.5, H_2O_2 - 1.72 g/l.15 min	100	[74]
	49 ng/l	Waste water	550 w/m^2, 17°C, pH2.5, H_2O_2 - 50 mg/l, 30 min	100	[123]

Modified from: Wang and Wang, [6].

Table S7 Removal efficiency of combined treatment technologies for pharmaceuticals

Pharmaceutical	UF	GAC	PAC	BAF	Ozone	PAC-UF	Ozone-BAF	UF-BAF
Azithromycin	No treatment	>80%	>80%	>80%	<20%	>80%	>80%	>80%
Ciprofloxacin		>80%	>80%	>80%	20-80%	>80%	>80%	>80%
Clarithromycin		>80%	>80%	>80%	<20%	>80%	>80%	>80%
Diclofenac		>80%	>80%	>80%	>80%	>80%	>80%	>80%
E2 (17β-estradiol)		>80%	>80%	>80%	>80%	>80%	>80%	>80%
EE2 (17α-ethinyl estradiol)		>80%	>80%	>80%	>80%	>80%	>80%	>80%
Erythromycin		>80%	>80%	>80%	<20%	>80%	>80%	>80%
Ibuprofen		>80%	>80%	>80%	20-80%	>80%	>80%	>80%
Carbamazepine		>80%	>80%	>80%	>80%	>80%	>80%	>80%
Levonorgestrel		>80%	>80%	>80%	>80%	>80%	>80%	>80%
Metoprolol		>80%	>80%	>80%	>80%	>80%	>80%	>80%
Oxazepam		>80%	>80%	>80%	20-80%	>80%	>80%	>80%
Propranolol		>80%	>80%	>80%	>80%	>80%	>80%	>80%
Sertraline		>80%	<20%	>80%	20-80%	<20%	>80%	>80%
Sulfamethoxazole		>80%	>80%	>80%	>80%	>80%	>80%	>80%
Trimethoprim		>80%	20-80%	>80%	>80%	20-80%	>80%	>80%

Adapted from: The Swedish Environmental Protection Agency [125].

Table S8 Technical assessment of combined treatment technologies for pharmaceuticals

	**UF**	**GAC**	**PAC**	**BAF**	**Ozone**	**PAC-UF**	**Ozone-BAF**	**UF-BAF**
Robustness	+++	+++	+++	++	+++	+++	+++	+++
Availability in full-scale	+++	+++	+++	+++	+++	not tested	+++	not tested
Easy to maintain	++							
Footprint	++	++	++	++	+++	++	++	++
Residue with disposal problem	no	no	yes	no	yes	yes	no	no

Adapted from: The Swedish Environmental Protection Agency [125].

Table S9 Comparison for MBR, OMBR and MDBR in wastewater treatment (Modified from Pathak et al. [93]

**Parameters/description**	**MBR**	**OMBR**	**MDBR**
Membrane type	Low-pressure MF/UF membranes are employed.<br>(MF MWCO = 1000 kDa)<br>(UF MWCO = 10 - 100 kDa)<br>Liquid (permeate water) inside the lumen.	Forward osmosis (FO) semi-permeable membranes are used.<br>FO (MWCO = 0,1 - 2 kDa)<br><br>Liquid (permeate water) inside the lumen.	Hydrophobic membrane distillation membrane (MD) is used.<br>MD (MWCO < 150 Dalton<br><br>Vapor phase (permeate) inside the lumen.
Removal mechanism	Size exclusion is principal removal mechanism.	Steric hindrance and electrostatic repulsion are principal removal mechanisms.	Steric hindrance is the principal removal mechanism.
Membrane process influence on economy	In MBR, the design has evolved to continually improve the economy of energy required for scouring, backwashing and aeration.	In OMBR, fine bubble diffusion for oxygen transfer and a longer interval between backwashing and cleaning should require less energy.	In MDBR, the waste heat source can be utilized, thus saving energy and minimizing GHG emission.<br>MD utilizes waste heat directly with a heat exchanger.
Energy consumption (kWh/m^3)	In MBRs total energy estimate is 4.2 kWh/m^3 water treated.	In OMBR total energy estimate is 2.8 kWh/m3 water treated.	Electrical energy requirement for RO would increase as feed solution salinity increases whereas MD is only minimally affected by feed solution salinity.
OMP removal	MF/UF membrane of the MBR process is commercialized.<br>Hydrophilic OMP removal is too low.	Membrane stability is major concern.<br>CTA membrane can operate in narrow pH range and stability of membrane due to biodegradation being a concern.	Complete rejection of inorganic salts and OMPs.<br>Ammonia and CO_2 can seep through MD membrane.

Table S10 Summary of recently published OMBR studies (Modified from Pathak et al. [93]

FO membrane	Draw solution	HRT (h)	SRT (d)	MLSS (g/l)	Water flux (LMH)	Bioreactor conductivity	OMP	Removal (%)	Ref
Plate-and-frame FO membrane Hydration Technology Innovations (HTI, USA) made of cellulose triacetate (CTA)	1 M NaCl	25.25	30	--	11.88	--	CBZ 100 (μg/L)	93.27 ±3.77%	Yao et al., [126]
HTI-CTA FO membrane	0.75 M NaCl	30	70	3.5	7–5.5	2.5 (g/L)	Caffeine<br>Atrazine<br>Atenolol	94<br>51<br>100	Pathak et al., [127]

Table S11 Summary of recently published OMBR studies

Feed Solution	MD Membrane	Temperature		HRT (h)	SRT (d)	MLSS (g/L)	Water flux (LMH)	DO (mg/L)	OMP	Removal (%)	Ref
		Feed °C	Permeate °C								
Synthetic wastewater	PTFE membrane	45	20	4 d	--	10	--	--*	Amtriptyline, Atrazine<br>Bisphenol A, Caffeine<br>Carazolol, Carbamazepine<br>Clozapine, Diazinon<br>Diclofenac, Diuron<br>Gemfibrozil, Ibuprofen<br>Ketoprofen, Linuron<br>Naproxen, Paracetamol<br>Phenylphenol, Primidone<br>Propylparaben, Simazine<br>Sulfamethoxazole, TCEP<br>Triamterene, Triclocarban<br>Triclosan, Trimethoprim	99, 74<br>85, 99<br>97, 90<br>99, 99<br>75, 99<br>99, 99<br>99, 93<br>97, 99<br>80, 99<br>91, 79<br>89, 92<br>98, 95<br>85, 99	Song et al., [128]
Synthetic wastewater	PTFE side stream	40	14	9.6 d		5.3	1.2	2.8	17α-Ethinylestradiol<br>17β-Estradiol<br>17β-Estrodiol-17- acetate<br>4-Tert-butyphenol<br>Ametryn, Amitriptyline<br>Atrazine, Benzophenone<br>Carbamazepine, Clofibric acid<br>Diclofenac, Estriol, Estrone<br>Fenoprop, Gemfibrozil<br>Ibuprofen, Ketoprofen<br>Naproxen, Octocrylene<br>Oxybenzone,<br>Pentachlorophenol<br>Primidone, Propoxure<br>Salicylic acid, Triclosan	99<br>100<br>100<br>98<br>99, 99<br>96, 97<br>96, 100<br>95, 98,<br>100<br>97, 98<br>100, 99<br>100, 97<br>99, 97<br>100, 100<br>96, 98	Wijekoon et al., [95]

Table S12 Comparison of the performance of four membrane processes: aerobic MBR, anaerobic MBR, biofilm MBR, and FO-MBR in terms of energy demand and their impact on climate change (modified from Krzeminski et al., [96] and Pathak et al, [93]).

Process type	Energy related emissions	Climate change impact
**MBR**	High	High
**AnMBR**	Low	High
**BF-MBR**	High/medium	High/medium
**FO-MBR**	Medium	Medium
